# *Mangifera indica* as propolis source: what exactly do bees collect?

**DOI:** 10.1186/s13104-021-05863-7

**Published:** 2021-12-13

**Authors:** Milena Popova, Boryana Trusheva, Nia Ilieva, Le Nguyen Thanh, Nguyen Thi Phuong Lien, Vassya Bankova

**Affiliations:** 1 Institute of Organic Chemistry with Centre of Phytochemistry, Bulgarian Academy of Sciences, Sofia, Bulgaria; 2 Institute of Marine Biochemistry and Graduate University of Science and Technology, Vietnam Academy of Science and Technology, Hanoi, Vietnam; 3 Institute of Ecology and Biological Resources, Vietnam Academy of Science and Technology, Hanoi, Vietnam

**Keywords:** Propolis, Stingless bees, *Apis mellifera*, *Mangifera indica*, Resin, Latex

## Abstract

**Objective:**

The mango tree *Mangifera indica* is known as one of the botanical sources of propolis in Tropical regions. There are two different materials which bees can collect from a mango tree to produce propolis: the resin of the tree bark, and the latex found on the fruits. We performed the study of the chemical profile of mango resin in comparison with propolis in order to clarify its importance as propolis source.

**Results:**

We compared the chemical profiles (by GC–MS analysis of ethanol extracts after silylation) of the resin and samples of propolis: of stingless bees (3 Vietnames, 2 Indonesian), and one of *Apis mellifera* from Thailand. In the resin and all propolis samples, 25 compounds were identified: fatty acids, cardanols (alk(en)yl phenols), cardols, anacardic acids, triterpene alcohols and ketones, cycloartane type triterpenic acids. All samples have the same qualitative composition but there are important quantitative differences. Considering literature data on mango latex, we conclude that bees of different species, make use of the two propolis sources offered by mango: bark resin and fruit latex, in different proportions. We also confirmed for the first time the presence of alk(en)yl phenols and anacardic acids in the tree bark resin of mango.

## Introduction

The mango tree *Mangifera indica* L. has been recognized as propolis botanical source in 2005 [1]. There are communications reporting propolis of both European honey bees *Apis mellifera* and stingles bees Meliponini, originating from mango trees in numerous tropical countries in Africa [2, 3], the Americas [4–6], Southeast Asia [7–9], and even Oceania [10]. These reports are based on the identification of mango chemical markers in the corresponding propolis samples. There are two structural groups of such markers: the cycloartane type triterpenes cycloartenol, mangiferolic, isomangiferolic, and ambolic acids; and the group of phenolic lipids, mainly cardols (alk(en)yl resorcinols) [11, 12].

Studying propolis, some authors have found the abovementioned cycloartanes (but not any phenolic lipids), and based on these findings they concluded that *Mangifera indica* L. was the main plant source of the studied samples [1, 8, 13, 14]. Others have identified only the cardols, no cycloartane acids, and made the same conclusion [4, 15]. In these cases, the respective compounds have been isolated, purified and identified by spectral methods. A third group of studies, which have applied chemical profiling by hyphenated techniques, report the identification of both groups of markers [2, 7, 9]. It turns out that mango propolis contains cycloartanes, as well as phenolic lipids.

Obviously, the source plant of this propolis type is confirmed to be *M. indica* beyond doubt. However, there are two different materials which bees can collect from a mango tree in order to produce propolis: the reddish-brown resin which appears on the tree bark, and the latex found on the fruits. Both materials have been chemically studied. The resin has been found to contain triterpenes, mainly cycloartanes [13], and cardols have also been reported [4]. The latex, on the other hand, contains monoterpenes with typical raw mango aroma [16, 17], cardols [18–20], carbohydrates, and small amounts of proteins [21, 22]. No triterpenes have been detected in latex, although several detailed studies have been carried out. So, we studied the chemical profile of mango resin in order to clarify its importance as propolis source.

## Main text

### Methods

#### Sample collection

Resin sample was collected in Mai Chau village, Hoa Binh province, designated as sample 1. Details on propolis samples are presented in Table 1.

**Table 1:** Propolis samples

Sample	Geographical origin	Bee species
2	Da Nang, Vietnam	*Lisotrigona cacciae*
3	Daklak, Vietnam	*Lisotrigona cacciae*
4	Hoa Binh, Vietnam	*Lepidotrigona* *ventralis*
5	Banten province, Indonesia^a^	*Tetragonula laeviceps*
6	South Kalimantan Province, Indonesia^a^	*Heterotrigona itama*
7	Phrae, Northern Thailand^b^	*Apis mellifera* L.

^a^Data from [23]

^b^Data from [9]

#### Sample preparation and GC–MS analysis

The resin sample 1 and crude propolis samples 2–4 were extracted with 70% ethanol (1:10, w/v) at room temperature for 24 h (2 times). After evaporation in vacuo, the dry extracts were silylated: about 5 mg dry extract was mixed with 50 μL of dry pyridine and 75 μL of N,O-bis(trimethylsilyl)trifluoroacetamide (BSTFA). The GC–MS analysis was performed with Hewlett-Packard 5890 series II Plus, linked to a Hewlett–Packard 5972 mass spectrometer system equipped with a 30 m DB-17HT capillary column, 0.25 mm i.d., 0.15 µm film thickness. The temperature program from 100 to 320 °C at a rate of 5 °C/min; carrier gas Helium at a flow rate of 0.8 mL/min. The split ratio was 75:1, the injector temperature 300 °C, and the ionization voltage 70 eV.

#### Compound identification and quantitation

The compounds identification was accomplished using commercial libraries, literature data, and/or comparison with mass spectra and retention times of reference compounds. The amounts of components of the propolis extracts were determined by considering their areas as the percentage of the total ion current. The ion currents generated depend on the characteristics of the compound and for this reason are regarded semiquantitative but allow comparison between samples.

### Results and discussion

**Fig. 1:**
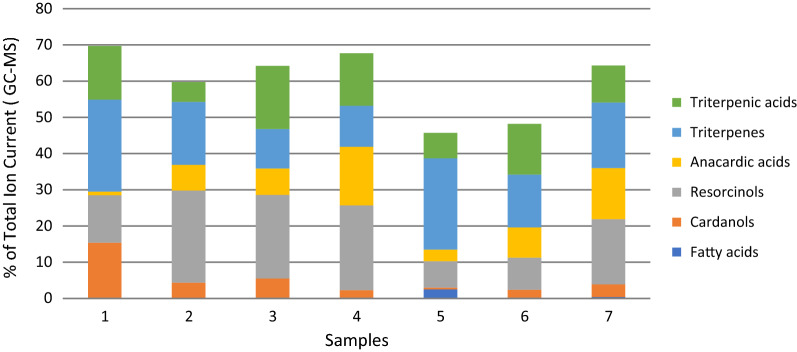
Chemical profiles of the studied resin and propolis samples

In order to clarify the role of mango tree bark resin as propolis source, we compared the chemical profiles (by GC–MS analysis of ethanol extract after silylation) of the resin and three samples stingless bees propolis from Vietnam. In the resin, 25 compounds were identified: 2 fatty acids, 6 cardanols (alk(en)yl phenols), 4 cardols (alk(en)yl resorcinols), 2 anacardic acids, 8 triterpene alcohols and ketones, and 3 cycloartane type triterpenic acids (Table 2). All these compounds were present in the Vietnamese stingless bees propolis samples. In all samples, the main cardanols were heptadecenyl and nonadecenyl phenol; major cardol was heptadecenyl resorcinol, and most abundant triterpenes were cycloartenol, mangiferolic and isomangiferolic acid. The results of the GC–MS analysis are represented on Fig. 1 for the abovementioned 6 groups of compounds.

**Table 2:** Compounds identified in all studied samples

Compound type	Compound
Fatty acids	
	Palmitic acid
	Stearic acid
Cardanols (alk(en)yl phenols)	
	Cardanol C_15:0_
	Cardanol C_15:1_
	Cardanol C_17:0_
	Cardanol C_17:1_
	Cardanol C_17:1_ (isomer)
	Cardanol C_19:1_
Cardols (alk(en)yl resorcinols)	
	Resorcinol C_15:0_
	Resorcinol C_17:0_
	Resorcinol C_17:1_
	Resorcinol C_19:1_
Anacardic acids	
	Anacardic acid C_19:0_
	Anacardic acid C_19:1_
Triterpenic alcohols and ketones	
	Lanosterol
	*β*-Amyrin
	Cycloartenol
	*α*-Amyrin
	Lupeol
	*β*-Amyrenone
	*α*-Amyrenone
	Lupenone
Cycloartane type triterpenic acids	
	Mangiferolic acid
	Ambolic acid
	Isomangiferolic acid

The results were compared with our data from earlier studies of Indonesian stingless bees propolis [23] and Thai *A. mellifera* propolis [9], classified as *Mangifera* type. The same 25 constituents have been identified in these samples, too. The quantitative data of these samples for the respective compound groups are also displayed in Fig. 1.

It is important to note that cardanols and anacardic acids were found in mango tree bark resin for the first time in our study. The resin, and propolis of different bee species and different locations have the same qualitative composition. However, there are quantitative differences and especially in the ratio phenolic lipids/triterpenes (PL/TT). This ratio is higher than 1 (1.2–1.6) in the three Vietnamese propolis samples and the Thai *A. mellifera* propolis. It is however 0.7 for the resin, and 0.5 and 0.7 for the Indonesian propolis. It is noteworthy that the Vietnamese propolis samples come from three distinct regions of the country but are similar in their profiles, and differ quantitatively from the resin profile. On the other hand, Indonesian samples are closer to the mango resin profile, although the resin was collected in Vietnam. One possible explanation could be the collection by bees of both the available mango exudates: the tree bark resin and the fruit latex, depending on their availability and bees’ preferences. In cases where the latex prevails, ratio PL/TT > 1 can be observed, while where PL/TT < 1 more resin than latex has been foraged by bees.

In conclusion, our study demonstrates that bees of different species, including stingless and *A. mellifera* bees, can make use of the two propolis sources offered by *Mangifera indica*, bark resin and fruit latex, in different proportions. We also confirmed for the first time the presence of alk(en)yl phenols and anacardic acids in the tree bark resin of mango.

## Limitations of the study

The mango resin sample was not collected in the vicinity of the bees’ nests. Another limitation is the lack of mango latex for comparative study.

## Data Availability

The dataset of the current study is available from the corresponding author on reasonable request.

## Abbreviations

BSTFA N: O-bis(trimethylsilyl)trifluoroacetamide
GC–MS: Gas chromatograpy–mass spectrometry
PL: Phenolic lipids
TT: Triterpenes
